# Multidimensional deep learning for grading and prognostic assessment of intrahepatic mass-forming cholangiocarcinoma

**DOI:** 10.1186/s13244-026-02350-0

**Published:** 2026-07-22

**Authors:** Liyong Zhuo, Wenjing Chen, Zijun Song, Lihong Xing, Xiaomeng Li, Jiawei Hao, Zimei Yang, Xuechun Wang, Caiying Li, Jianing Wang, Xiaoping Yin

**Affiliations:** 1 Department of Radiology, Affiliated Hospital of Hebei University, Baoding, People’s Republic of China; 2 Department of Research and Development, United Imaging Intelligence (Beijing) Co., Ltd., Beijing, People’s Republic of China; 3 Department of Critical Care Medicine, Baoding First Central Hospital, Baoding, People’s Republic of China; 4 Department of Research and Development, United Imaging Intelligence, Shanghai, People’s Republic of China; 5 Department of Medical Imaging, The Second Hospital of Hebei Medical University, Shijiazhuang, People’s Republic of China

**Keywords:** Cholangiocarcinoma, Deep learning, Neoplasm grading, Magnetic resonance imaging, Prognosis

## Abstract

**Objective:**

To develop an interpretable magnetic resonance imaging (MRI)-based framework for preoperative histologic grading of intrahepatic mass-forming cholangiocarcinoma (IMCC) and exploratory prognostic stratification.

**Materials and methods:**

A retrospective analysis was conducted on preoperative MRI from 333 IMCC patients across three centers (training cohort, *n* = 240; external validation cohort, *n* = 93). An ensemble deep learning (DL) framework synergizing 2.5D and 3D ResNet-50 architectures was constructed. Significant variables from clinical-laboratory-imaging (ClinLabImag) features, radiomics, and DL outputs were integrated into a combined model. Discrimination was assessed using the area under the receiver operating characteristic curve (AUC). Model interpretability was evaluated with Gradient-weighted Class Activation Mapping (Grad-CAM) and SHapley Additive exPlanations (SHAP), and the Kaplan–Meier method was used to compare overall survival (OS) between risk groups.

**Results:**

The DL model achieved an external validation AUC of 0.804 (95% confidence interval (CI): 0.712–0.896), significantly outperforming standalone 2.5D (*p* = 0.025) and 3D architectures (*p* = 0.030). The Combined model (AUC: 0.843 [95% CI, 0.749–0.938]) showed better external validation performance than the Radiomics (*p* = 0.019) and ClinLabImag models (*p* = 0.017), with only modest, non-significant improvement over the DL model (*p* = 0.355). SHAP analysis showed that DL features contributed most to model predictions. The Combined model showed exploratory OS differences between risk groups.

**Conclusion:**

An interpretable multidimensional MRI-based DL framework supports noninvasive preoperative grading in IMCC and provides exploratory prognostic information.

**Critical relevance statement:**

This study critically evaluates an interpretable multidimensional MRI-based deep learning framework for preoperative grading and exploratory prognostic stratification of intrahepatic mass-forming cholangiocarcinoma, supporting individualized radiologic risk assessment before treatment.

**Key Points:**

- Reliable noninvasive MRI biomarkers are needed for preoperative grading and prognostic assessment of intrahepatic mass-forming cholangiocarcinoma to guide individualized treatment planning.
- The combined multidimensional MRI model achieved favorable external validation performance and showed exploratory overall survival differences between IMCC risk groups.

**Graphical Abstract:**

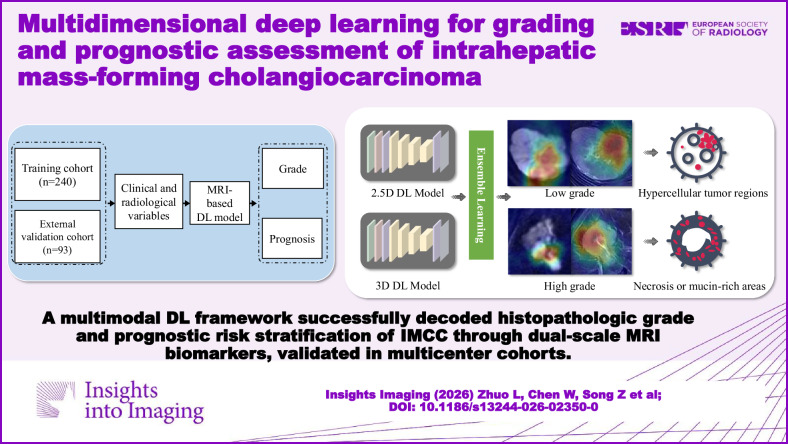

## Introduction

Classified as the second most common primary liver cancer after hepatocellular carcinoma, intrahepatic cholangiocarcinoma has shown a 140% increase in global incidence over the past four decades [1]. The most common subtype, intrahepatic mass-forming cholangiocarcinoma (IMCC), accounts for approximately 60% of intrahepatic cholangiocarcinoma cases and is characterized by rapid tumor expansion, early dissemination, and poor clinical outcomes [2].

Preoperative grading of IMCC is essential for personalized treatment planning. Distinguishing high-grade tumors (associated with < 15% 5-year survival) from low-grade tumors (> 40% 5-year survival) [3] can guide intensified neoadjuvant therapy or support decisions to avoid futile surgical exploration in unresectable disease [4]. Tumor grade also influences recommendations regarding surgical margins and adjuvant therapy [5]. Histologically, higher grades are associated with pronounced desmoplasia, perineural invasion, and immunosuppressive tumor microenvironments [6]. However, preoperative biopsy-based grading is limited by sampling error—discordance rates up to 21.5% have been reported in mucin-rich tumors [7]—underscoring the need for robust noninvasive imaging biomarkers [8].

Multiparametric magnetic resonance imaging (MRI), particularly diffusion-weighted imaging (DWI) and T2-weighted imaging (T2WI), is central to IMCC assessment [9]. DWI reflects cellular density and extracellular matrix composition, whereas T2WI delineates anatomical and ductal structures [10], providing a biologically meaningful foundation for advanced computational models.

Radiomics enables high-throughput quantitative feature extraction for tissue characterization [11], yet handcrafted features fail to capture complex spatial-volumetric traits such as infiltrative margins [12] or 3D spiculation [13]. Deep learning (DL) performs automated hierarchical feature learning from images [14], but single-architecture approaches remain limited in characterizing spatial-volumetric attributes [15]. Two-dimensional models capture intralesional heterogeneity yet incompletely represent volumetric growth patterns [16], while three-dimensional networks prioritize whole-tumor context at the expense of cellular-level detail [17, 18]. Transformer-based and attention-driven frameworks underscore the potential of long-range dependency modeling and multimodal fusion [19, 20].

Consistent with recent multimodality medical image fusion frameworks that combine complementary information from different imaging sources [21, 22], our study proposes an interpretable ensemble DL framework that integrates multidimensional MRI-based features for the preoperative prediction of histological grades in IMCC. We also explored whether model-defined risk groups were associated with overall survival (OS) differences, while hypothesizing that the multidimensional MRI framework would improve grade discrimination compared with conventional imaging-based strategies.

## Materials and methods

### Participants

This study was conducted and reported in accordance with the Checklist for Artificial Intelligence in Medical Imaging guidelines [23]. It was approved by the institutional review boards of all participating centers (approval number: HDFYLL-KY-2024-021), and written informed consent was waived for all subjects. Preoperative multiparametric MRI from 333 patients with histologically confirmed IMCC between January 2018 and June 2024 were included. Patients with prior anticancer treatment, > 14 days between MRI and surgery/biopsy, or nondiagnostic image quality were excluded. The training cohort comprised 240 patients from two centers; an independent external validation cohort included 93 patients from a third center (Fig. S1).

### Histopathologic assessment and follow-up

Histopathological analysis was performed by two senior pathologists from the three participating institutions, blinded to MRI findings. Tumors were independently graded according to the 2019 World Health Organization (WHO) classification criteria [24] as low-, medium-, or high-grade; discordant cases were resolved by consensus. The histologic grades were dichotomized into low-grade (well-differentiated) and high-grade (moderately and poorly differentiated) based on prior evidence [25] showing similar poorer prognoses [26] for moderately and poorly differentiated IMCC as compared to well-differentiated tumors, as well as to address the limited sample size for balanced model development. OS, the sole survival endpoint, was calculated from the date of surgery (or biopsy in unresectable cases) to death from any cause or last follow-up. The administrative censoring date was 31 December 2024.

### MRI acquisition and image analysis

MRI scans were acquired using multiple vendors and field strengths: for the training dataset, GE 3.0 T (Discovery MR 750), United Imaging 1.5 T (uMR 588), and Siemens 1.5 T (Amira); for the external test dataset, GE 1.5 T (Optima MR360) and GE 3.0 T (Signa HDx). Detailed acquisition parameters are provided in Supplementary Table S1. Twelve qualitative imaging features were independently evaluated by two abdominal radiologists, with excellent interobserver agreement indicated by intraclass correlation coefficients ranging from 0.83 to 0.95. These included the targetoid sign, tumor thrombus, vascular traversal sign, intrahepatic duct dilatation, capsular retraction, enlarged lymph nodes, tumor margin, hepatic capsule retraction, T2WI signal pattern, DWI signal pattern, vascular involvement, and tumor size (full definitions in Supplementary Material Section 1). Discordant cases were adjudicated through joint reviewer consensus.

### Tumor segmentation and reproducibility analysis

Tumor volumes of interest (VOIs) were manually segmented independently on T2WI and DWI without cross-sequence reference using ITK-SNAP (v4.0.0) by two radiologists who were blinded to histopathological grades and patient outcome data, followed by consensus review and validation by a senior radiologist (> 15 years of experience), and were subsequently registered for alignment (Supplementary Section 4). Excellent inter- and intra-observer reproducibility was achieved (interobserver Dice 0.86 ± 0.05; full details in Supplementary Material Section 2).

### DL model construction

**Fig. 1:**
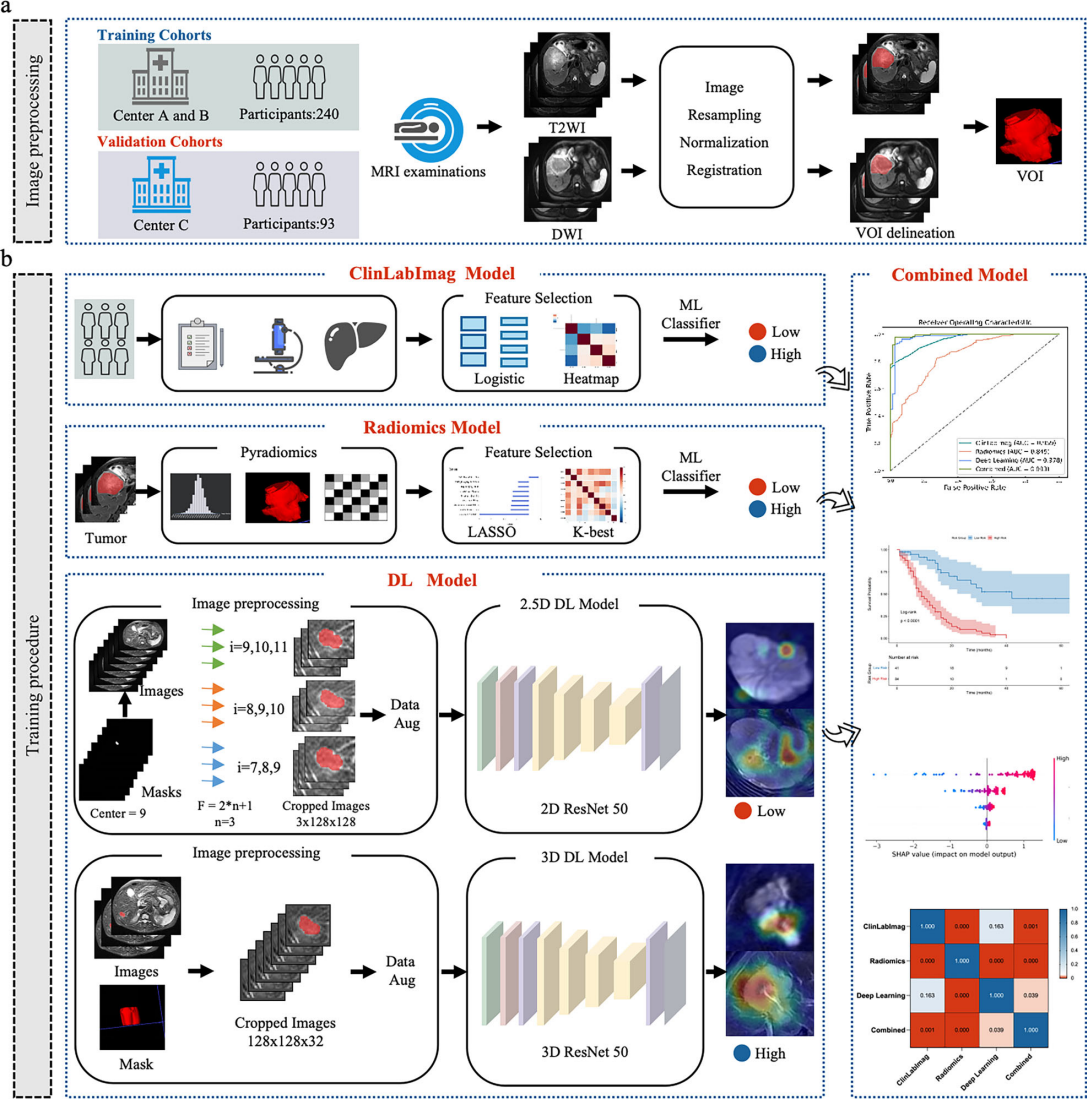
Multicenter image preprocessing and model development workflow. **a** Patient cohorts, MRI acquisition, image preprocessing, and VOI delineation. **b** Development of the ClinLabImag, radiomics, deep learning, and combined models, followed by performance, survival, and interpretability analyses. VOI, volume of interest; ClinLabImag, clinical-laboratory-imaging; ML, machine learning; DL, deep learning; Aug, augmentation; LASSO, least absolute shrinkage and selection operator

An ensemble deep learning framework integrating 2.5D and 3D ResNet-50 architectures was developed to process T2WI and DWI with standardized preprocessing (details in Supplementary Material Section 3, Fig. 1).

### Model training

Both frameworks employed identical training protocols. A batch size of 32 preprocessed images per batch was dynamically augmented using PyTorch’s DataLoader. To handle the imbalance between low- and high-grade IMCC, we adopted the Asymmetric Loss Single Label (ASLSingleLabel), a focal-like loss function optimized for class imbalance with focal modulation parameters configured as γ_pos = 0, γ_neg = 4 and a label smoothing coefficient of α = 0.1, together with extensive on-the-fly data augmentation (random grayscale transformation, rotation, flipping, and scaling) during training. Training utilized the AdamW optimizer with an initial learning rate of 1e-5, following a warmup cosine annealing schedule over 120 epochs. To mitigate overfitting, we applied multiple regularization strategies, including weight decay in AdamW, dropout layers in convolutional and fully connected blocks, batch normalization, and extensive on-the-fly data augmentation (random grayscale transformation, rotation, flipping, and scaling). The area under the receiver operating characteristic curve (AUC) was computed on the internal validation set after each epoch, and an early stopping strategy was adopted so that the checkpoint with the highest validation AUC was retained as the final model.

Architecture-specific configurations were as follows: (1) 2.5D ResNet-50: Three-channel input (stacked adjacent slices) fed into a standard ResNet-50 backbone; (2) 3D ResNet-50: Pretrained weights derived from 23 medical imaging datasets [27] were transferred, and the final layers were fine-tuned using study-specific data.

### Ensemble model integration

Probabilistic outputs from the 2.5D and 3D frameworks were fused using logistic regression. The ensemble combined discriminative information from both DWI and T2WI sequences, finalizing predictions through a weighted probabilistic fusion approach.

### Model construction

The optimal probability threshold for binary classification (low- vs high-grade) was determined using the Youden index on the training cohort’s receiver operating characteristic (ROC) curve and applied unchanged to the external validation cohort. No alternative thresholds were explored, prioritizing the prespecified balanced performance metric. Using Python 3.10.6, four final predictive models were constructed with logistic regression (workflow illustrated in Fig. 1): (1) ClinLabImag model, incorporating clinical, laboratory, and qualitative imaging features; (2) Radiomics model, based on selected radiomics features; (3) DL model, using the ensemble DL probability as the sole predictor; and (4) Combined model, integrating the ensemble DL probability, the 24 radiomics features, and the multivariable-significant ClinLabImag features. Detailed ClinLabImag, Radiomics construction procedures are provided in the Supplementary Material.

### Model interpretability

Model interpretability was evaluated using Gradient-weighted Class Activation Mapping (Grad-CAM) and SHapley Additive exPlanations (SHAP). Two blinded abdominal radiologists independently rated Grad-CAM-to-VOI overlap on a 5-point Likert scale (Supplementary Sections 6 for full Grad-CAM validation).

### Statistical analysis

Continuous variables were expressed as medians with interquartile ranges (IQRs), and categorical variables as frequencies with percentages. Training-validation cohort comparisons utilized Fisher’s exact, Pearson χ² test, and Mann–Whitney U tests based on data characteristics. Differences in training dataset patient characteristics were examined through univariable and multivariable logistic regression (LR) analyses.

Predictive performance was evaluated using the AUC with 95% confidence intervals, decision curve analysis (DCA), and metrics including recall, specificity, accuracy, precision, and F1-score. Shapley Additive Explanations Analysis (SHAP) analysis [28] quantified the influence of characteristics on model predictions. Survival outcomes were evaluated using Kaplan–Meier analysis with comparisons made via the log-rank test. Statistical computations were performed using Python (version 3.10.6), adopting a two-tailed *p* < 0.05 significance threshold.

## Results

### Patient characteristics

The multicenter study ultimately enrolled 333 IMCC patients across three institutions (Fig. S1). The training cohort comprised 240 cases from Center A (*n* = 148) and Center B (*n* = 92), exhibiting a median age of 60 years (IQR: 55–66 years). External validation involved 93 patients from Center C with comparable age distribution (median 59 years, IQR: 55–65 years).

Significant inter-cohort disparities were identified between training and external validation cohorts, spanning clinical, laboratory, and imaging characteristics. The cohorts diverged in sex distribution (*p* < 0.001) and carbohydrate antigen 125 (CA-125) levels (*p* < 0.001). Imaging signatures demonstrated significant heterogeneity across multiple parameters: lesion localization (*p* < 0.001), tumor thrombus (*p* < 0.001), morphological abnormalities (tumor margin: *p* < 0.001; hepatic capsule retraction: *p* = 0.030; T2WI tumor boundary: *p* = 0.001;) signal patterns (targetoid sign on T2WI: *p* < 0.001; targetoid sign on DWI: *p* = 0.005), enlarged lymph nodes (*p* < 0.001), vascular traversal sign (*p* = 0.003). Detailed patient characteristics are provided in Table 1.

**Table 1:** Comparative analysis of clinical, laboratory, and imaging features in the training and validation cohorts

Characteristic	Training cohorts (*n* = 240)			Validation cohorts (*n* = 93)			*p-*inter value
	Low grade (*n* = 77)	High grade (*n* = 163)	*p*-intra value	Low grade (*n* = 21)	High grade (*n* = 72)	*p*-intra value	
Age (years)	61.000 (54.000, 69.000)	58.000 (52.000, 65.000)	0.053	58.000 (54.000, 63.000)	60.000 (54.750, 67.000)	0.669	0.293
Sex			0.133			0.209	< 0.001*
Male	23 (29.870)	65 (39.877)		17 (80.952)	48 (66.667)		
Female	54 (70.130)	98 (60.123)		4 (19.048)	24 (33.333)		
CA199			0.079			0.043*	0.272
≤ 39	27 (35.065)	41 (25.153)		12 (57.143)	21 (29.167)		
> 39	26 (33.766)	47 (28.834)		6 (28.571)	24 (33.333)		
CEA			0.038*			0.131	0.172
≤ 4.7	29 (37.662)	58 (35.583)		13 (61.905)	31 (43.056)		
> 4.7	24 (31.169)	30 (18.405)		5 (23.810)	14 (19.444)		
AFP			0.015*			0.033*	0.111
≤ 7	44 (57.143)	73 (44.785)		11 (52.381)	46 (63.889)		
> 7	14 (18.182)	19 (11.656)		5 (23.810)	3 (4.167)		
CA125			0.248			0.020*	< 0.001*
≤ 35	20 (25.974)	28 (17.178)		13 (61.905)	31 (43.056)		
> 35	16 (20.779)	33 (20.245)		5 (23.810)	8 (11.111)		
Lesion location			0.073			0.769	< 0.001*
Left lobe	16 (20.779)	59 (36.196)		9 (42.857)	39 (54.167)		
Right lobe	36 (46.753)	66 (40.491)		11 (52.381)	29 (40.278)		
Both	25 (32.468)	38 (23.313)		1 (4.762)	4 (5.556)		
Tumor size (cm)							
	3.230 (1.960–4.100)	8.90 (7.740–9.950)	< 0.001*	2.830 (1.970–5.110)	8.730 (7.460–10.100)	< 0.001*	0.358
Tumor margin			0.286			0.209	< 0.001*
Smooth	41 (53.247)	102 (62.577)		4 (19.048)	24 (33.333)		
Infiltrative	36 (46.753)	61 (37.423)		17 (80.952)	48 (66.667)		
T2WI tumor boundary			0.303			0.245	0.001*
Clear	38 (49.351)	92 (56.442)		5 (23.810)	27 (37.500)		
Blurry	39 (50.649)	71 (43.558)		16 (76.190)	45 (62.500)		
Bile duct dilatation			0.998			0.089	0.469
Absent	43 (55.844)	91 (55.828)		16 (76.190)	40 (55.556)		
Present	34 (44.156)	72 (44.172)		5 (23.810)	32 (44.444)		
Hepatic capsule retraction			0.780			0.557	0.030*
Absent	52 (67.532)	113 (69.325)		16 (76.190)	59 (81.944)		
Present	25 (32.468)	50 (30.675)		5 (23.810)	13 (18.056)		
Intrahepatic bile duct stones			0.584			0.440	0.376
Absent	73 (94.805)	157 (96.319)		21 (100.000)	70 (97.222)		
Present	4 (5.195)	6 (3.681)		0 (0.000)	2 (2.778)		
Tumor thrombus			0.039*			0.440	< 0.001*
Absent	45 (58.442)	117 (71.779)		21 (100.000)	70 (97.222)		
Present	32 (41.558)	46 (28.221)		0 (0.000)	2 (2.778)		
Vascular traversal sign			0.276			0.314	0.003*
Absent	54 (70.130)	125 (76.687)		20 (95.238)	63 (87.500)		
Present	23 (29.870)	38 (23.313)		1 (4.762)	9 (12.500)		
Enlarged lymph nodes			0.664			0.897	< 0.001*
Absent	62 (80.519)	135 (82.822)		12 (57.143)	40 (55.556)		
Present	15 (19.481)	28 (17.178)		9 (42.857)	32 (44.444)		
T2WI signal pattern			< 0.001*			< 0.001*	0.396
Targetoid sign	63 (81.818)	29 (17.791)		16 (72.727)	15 (21.127)		
No-targetoid sign	15 (18.182)	134 (82.209)		6 (27.273)	56 (78.873)		
DWI signal pattern			0.598			0.309	0.005*
Targetoid sign	46 (59.740)	92 (56.442)		7 (33.333)	33 (45.833)		
No-targetoid sign	31 (40.260)	71 (43.558)		14 (66.667)	39 (54.167)		

Categorical variables are expressed as frequency with percentages in parentheses. Qualitative variables are analyzed using Pearson’s χ^2^ test or Fisher’s exact test as appropriate, and quantitative variables are analyzed using the Mann–Whitney U-test. *p*-intra is the result of univariate analyses between the high-grade and low-grade groups; *p*-inter value represents the comparisons of characteristics between training and external validation sets

*CA199* carbohydrate antigen 199, *CEA* carcinoembryonic antigen, *AFP* α-fetoprotein, *CA125* carbohydrate antigen 12-5, *DWI* diffusion-weighted imaging, *T2WI* T2-weighted imaging

* *p* < 0.05

### Key variables for pathological grading

Univariate logistic regression analysis identified tumor size (OR = 8.512; 95% CI: 4.874–14.864; *p* < 0.001), tumor margin (OR = 0.739; 95% CI: 0.435–1.254; *p* = 0.026), vascular involvement (OR = 0.513; 95% CI: 0.296–0.889; *p* = 0.017), and T2WI signal pattern (OR = 1.700; 95% CI: 1.172–2.433; *p* = 0.004) as significant correlates of high-grade pathology (Table 2). In multivariable regression, tumor size (OR = 8.862; 95% CI: 4.936–15.911; *p* < 0.001), tumor margin (OR = 0.313; 95% CI: 0.100–0.978; *p* = 0.046), vascular involvement (OR = 0.265; 95% CI: 0.085–0.821; *p* = 0.021), and presence of targetoid sign on T2WI (OR = 1.628; 95% CI: 1.074–2.467; *p* = 0.022) emerged as independent predictors.

**Table 2:** Univariate and multivariable logistic regression analysis of the relationship between pathological grading and patient characteristics

	Univariate analysis			Multivariable analysis		
Variables	Odds ratio	95% CI	*p*-value	Odds ratio	95% CI	*p*-value
Age (years)	0.732	0.553–0.968	0.055			
Sex	0.808	0.611–1.068	0.134			
CA199	1.063	0.762–1.484	0.717			
CEA	0.868	0.662–1.138	0.306			
AFP	1.017	0.728–1.420	0.921			
CA125	1.139	0.868–1.496	0.348			
Tumor size	8.512	4.874–14.864	< 0.001*	8.862	4.936–15.911	< 0.001*
Lesion location	0.730	0.554–1.962	0.154			
Tumor margin	0.739	0.435–1.254	0.026*	0.313	0.100–0.978	0.046*
T2WI tumor boundary	0.868	0.662–1.137	0.304			
Bile duct dilatation	1.143	0.816–1.601	0.436			
Hepatic capsule retraction	1.022	0.732–1.428	0.898			
Intrahepatic bile duct stones	0.941	0.684–1.294	0.709			
Vascular involvement	0.513	0.296–0.889	0.017*	0.265	0.085–0.821	0.021*
Vascular traversal sign	0.863	0.663–1.125	0.277			
Enlarged lymph nodes	1.072	0.763–1.507	0.687			
DWI signal pattern	1.107	0.834–1.470	0.482			
T2WI signal pattern	1.700	1.172–2.433	0.004*	1.628	1.074–2.467	0.022*

*CI* confidence interval, *CA19-9* carbohydrate antigen 19-9, *CEA* carcinoembryonic antigen, *AFP* α-fetoprotein, *CA125* carbohydrate antigen 12-5, *DWI* diffusion-weighted imaging, *T2WI* T2-weighted imaging

* *p* < 0.05

Feature selection reduced the initial 4528 radiomics features extracted from T2WI and DWI sequences to 24 key predictors for modeling pathological grade (Table S2). To enhance biological interpretability, we visualized the 10 most influential DL-derived features and 10 radiomics features in a heatmap (Supplementary Fig. S2).

### Performance disparities across dimensional DL architectures

**Fig. 2:**
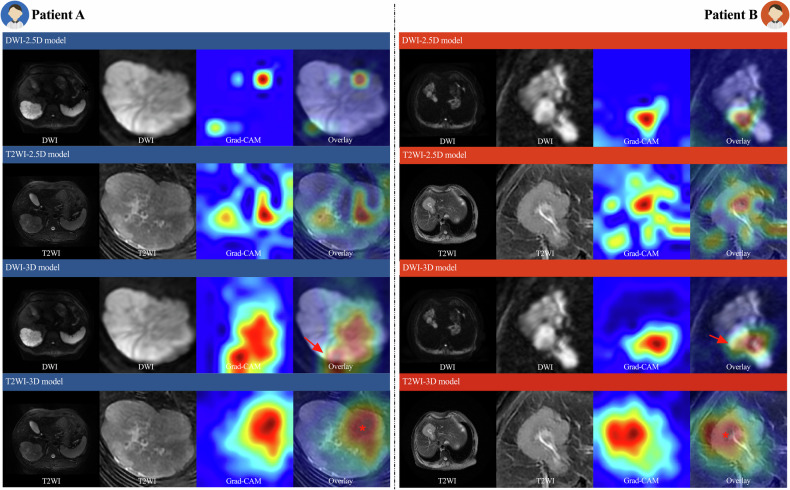
Gradient-weighted Class Activation Mapping (upper/lower: 2.5D/3D models) visually interprets DL model decision-making by prioritizing diagnostically critical regions on DWI and T2WI. Comparative visualization between low-grade (patient A) and high-grade (patient B) intrahepatic mass-forming cholangiocarcinoma reveals enhanced spatial reasoning in 3D models—DWI highlights tumor cell-dense regions (arrows), while T2WI maps necrotic cores (asterisks)—providing radiologists with pathophysiology-aligned explanations to augment clinical trust. Color gradient: blue (low relevance) to red (high attention). DWI, diffusion-weighted imaging; T2WI, T2-weighted imaging

In 3D frameworks, DWI emphasized hyperintense tumor regions (indicated by arrows), whereas T2WI accentuated free water-dominant zones (indicated by asterisks, Fig. 2).

**Fig. 3:**
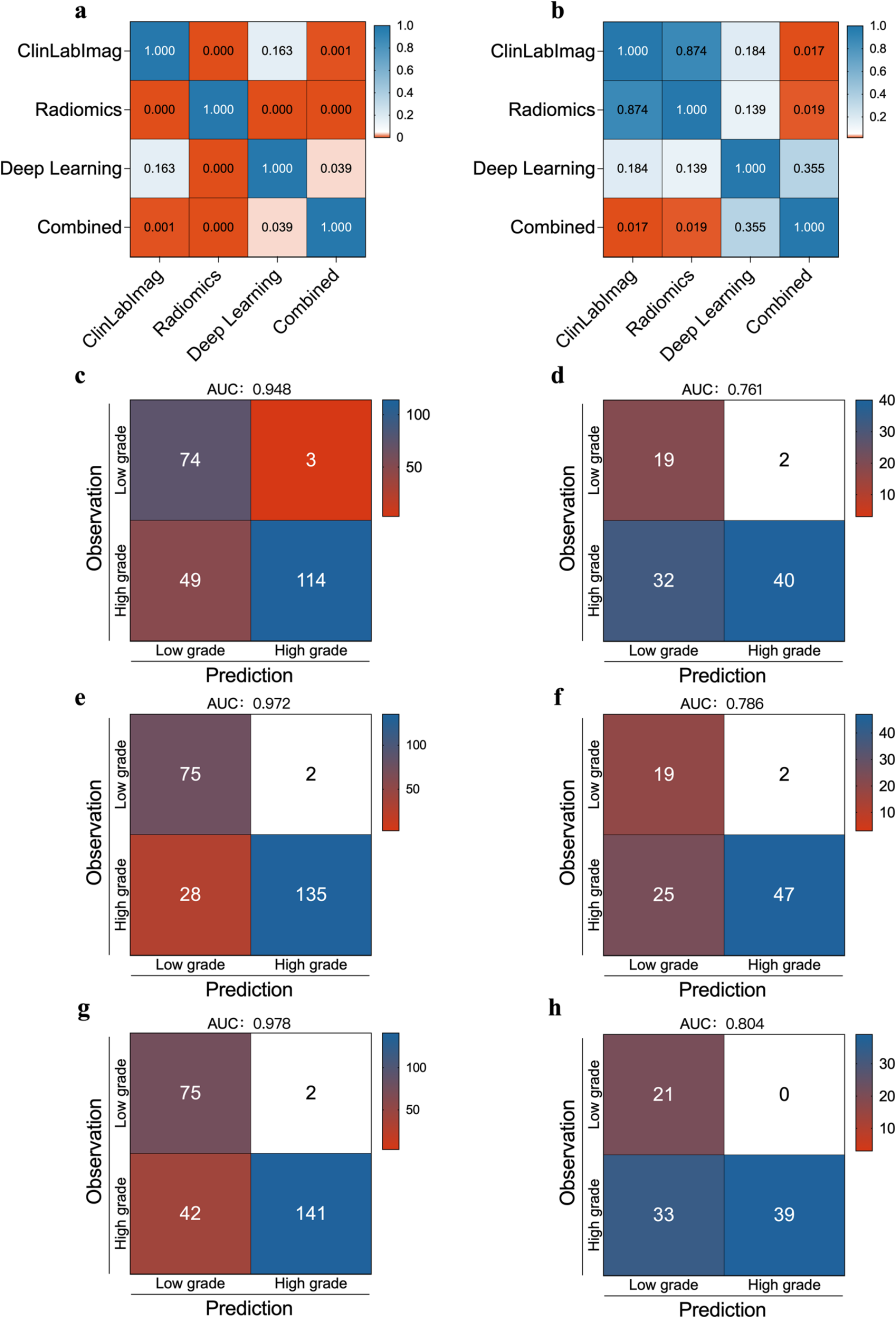
Model performance evaluation for intrahepatic cholangiocarcinoma grading. Delong test heatmaps compare four models (ClinLabImag, Radiomics, Deep Learning, Combined) on training and external validation cohorts (**a**, **b**). Confusion matrices illustrate classification performance with corresponding AUC values for: 2.5D models (**c**, **d**), 3D models (**e**, **f**), and ensemble DL models (**g**, **h**). AUC, area under the receiver operating characteristic curve

The ensemble DL model, which integrates probabilities from both 2.5D and 3D architectures, achieved superior diagnostic performance (Table 3, Fig. 3c–h). In external validation, it attained the highest AUC (0.804 [95% CI: 0.712–0.896]), significantly outperforming standalone 2.5D (net reclassification improvement (NRI) *Z* = 2.242, *p* = 0.025) and 3D frameworks (NRI *Z* = 2.169, *p* = 0.030).

**Table 3:** Performance disparities across dimensional architectures: 2.5D vs 3D vs Ensemble DL for IMCC grading

Models	AUC (95% CI)	Recall	Specificity	Accuracy	Precision	F1 score
2.5D DL framework						
Training cohorts	0.948 (0.922–0.975)	0.699	0.961	0.783	0.974	0.814
External validation cohorts	0.761 (0.657–0.866)	0.556	0.905	0.634	0.952	0.702
3D DL framework						
Training cohorts	0.972 (0.952–0.993)	0.828	0.974	0.875	0.985	0.900
External validation cohorts	0.786 (0.679–0.894)	0.653	0.905	0.710	0.959	0.777
DL model						
Training cohorts	0.978 (0.958–0.998)	0.748	0.974	0.821	0.984	0.850
External validation cohorts	0.804 (0.712–0.896)	0.556	1	0.656	1	0.714

*CI* confidence interval, *AUC* area under the receiver operating characteristic curve, *DL* deep learning

### Generalization in external validation

**Fig. 4:**
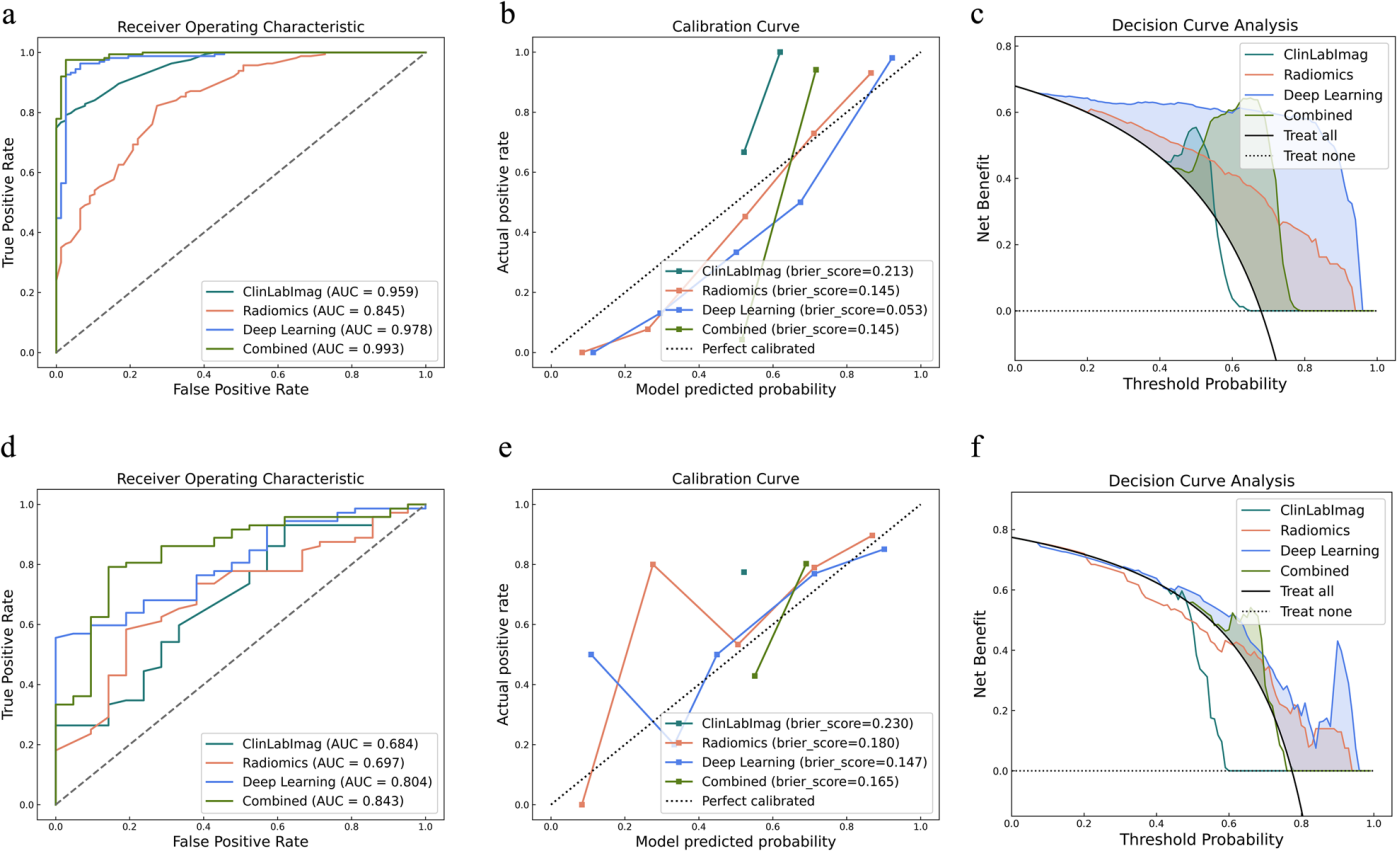
The receiver operating characteristic curves (**a**, **d**), calibration curves (**b**, **e**), and decision curves (**c**, **f**) of the different models in the training cohort and validation cohort

Diagnostic performance metrics across the four prediction models are detailed in Table 4 and Fig. 4. The Combined model achieved the highest external validation performance with an AUC of 0.843 (95% CI: 0.749–0.938). It concurrently demonstrated optimal precision (0.934 vs Radiomics’ 0.902) and the highest F1 score in the external validation cohort (0.857 vs DL’s 0.714). DeLong’s test showed no significant difference between the Combined model and the DL model (*p* = 0.355), whereas the Combined model significantly outperformed the ClinLabImag (*p* = 0.017) and Radiomics models (*p* = 0.019, Fig. 3b).

**Table 4:** Discrimination performance comparison of the prediction models for IMCC grading

Models	AUC (95% CI)	Recall	Specificity	Accuracy	Precision	F1 score
ClinLabImag model						
Training cohorts	0.959 (0.938–0.979)	1	0.416	0.812	0.784	0.879
External validation cohorts	0.684 (0.554–0.814)	0.931	0.381	0.806	0.838	0.882
Radiomics model						
Training cohorts	0.845 (0.793–0.897)	0.626	0.831	0.692	0.887	0.734
External validation cohorts	0.697 (0.574–0.820)	0.514	0.810	0.581	0.902	0.655
DL model						
Training cohorts	0.978 (0.958–0.998)	0.748	0.974	0.821	0.984	0.850
External validation cohorts	0.804 (0.712–0.896)	0.556	1	0.656	1	0.714
Combined model						
Training cohorts	0.993 (0.985–1)	0.963	0.974	0.967	0.987	0.975
External validation cohorts	0.843 (0.749–0.938)	0.792	0.810	0.796	0.934	0.857

The Combined model denotes the full integrated model that includes the DL probability, selected radiomics features, and ClinLabImag variables

*CI* confidence interval, *AUC* area under the receiver operating characteristic curve, *DL* deep learning

DCA showed that the Combined model provided the highest net benefit at threshold probabilities of 0.2–0.8 (Fig. 4f), with significant advantages over ClinLabImag (*p* = 0.025) and DL (*p* = 0.004) models, but no significant difference from the Radiomics model (*p* = 0.744). The DL model demonstrated greater net benefit within the 0.1–0.4 range, significantly outperforming Radiomics (*p* = 0.002) and ClinLabImag (*p* < 0.001) models.

### Model interpretability

**Fig. 5:**
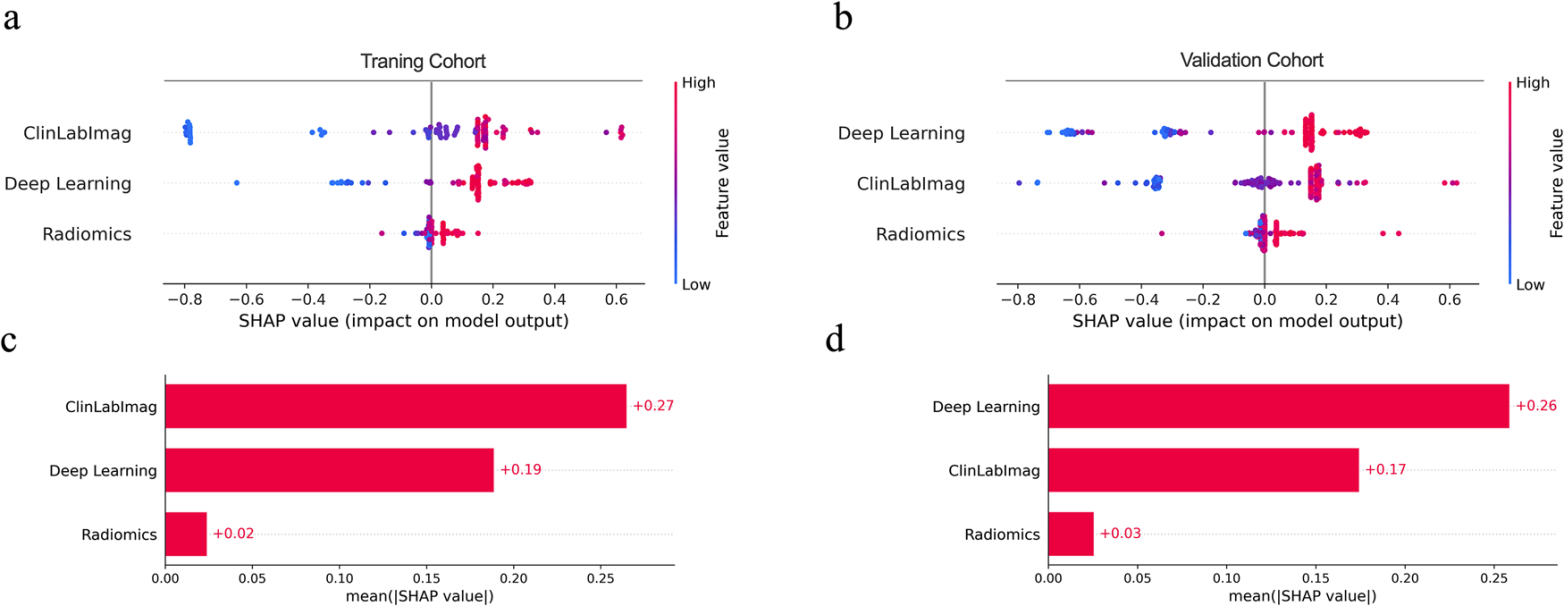
SHAP-based interpretation of feature contributions in training and validation cohorts. SHAP summary plots showing the impact of Deep Learning, ClinLabImag, and Radiomics features in training (**a**) and validation (**b**) cohorts. Mean absolute SHAP values indicating overall feature importance in training (**c**) and validation (**d**) cohorts. ClinLabImag, clinical-laboratory-imaging

At the global level (Fig. 5b), SHAP summary plots indicated that DL-derived features dominated the overall predictive contribution (72.1%), followed by ClinLabImag features (21.6%) and Radiomics variables (6.3%). DL features exhibited the highest mean absolute SHAP value (+0.26), followed by ClinLabImag variables (+0.17) and radiomics features (+0.03).

At the local level (Fig. S3), SHAP force plots illustrated case-specific interpretability. Panels (a, c) represent low-grade IMCC cases dominated by negative DL and Radiomics contributions, whereas panels (b, d) show high-grade cases characterized by positive DL and Radiomics shifts. Panel (e) illustrates the feature contributions for each patient in the validation cohort. Each patient is plotted along the x-axis, while the magnitude and direction of feature contributions are represented by the red (high-grade) and blue (low-grade) segments; a larger red portion for an individual indicates a higher predicted grade.

### Prognostic evaluation of the prediction model

**Fig. 6:**
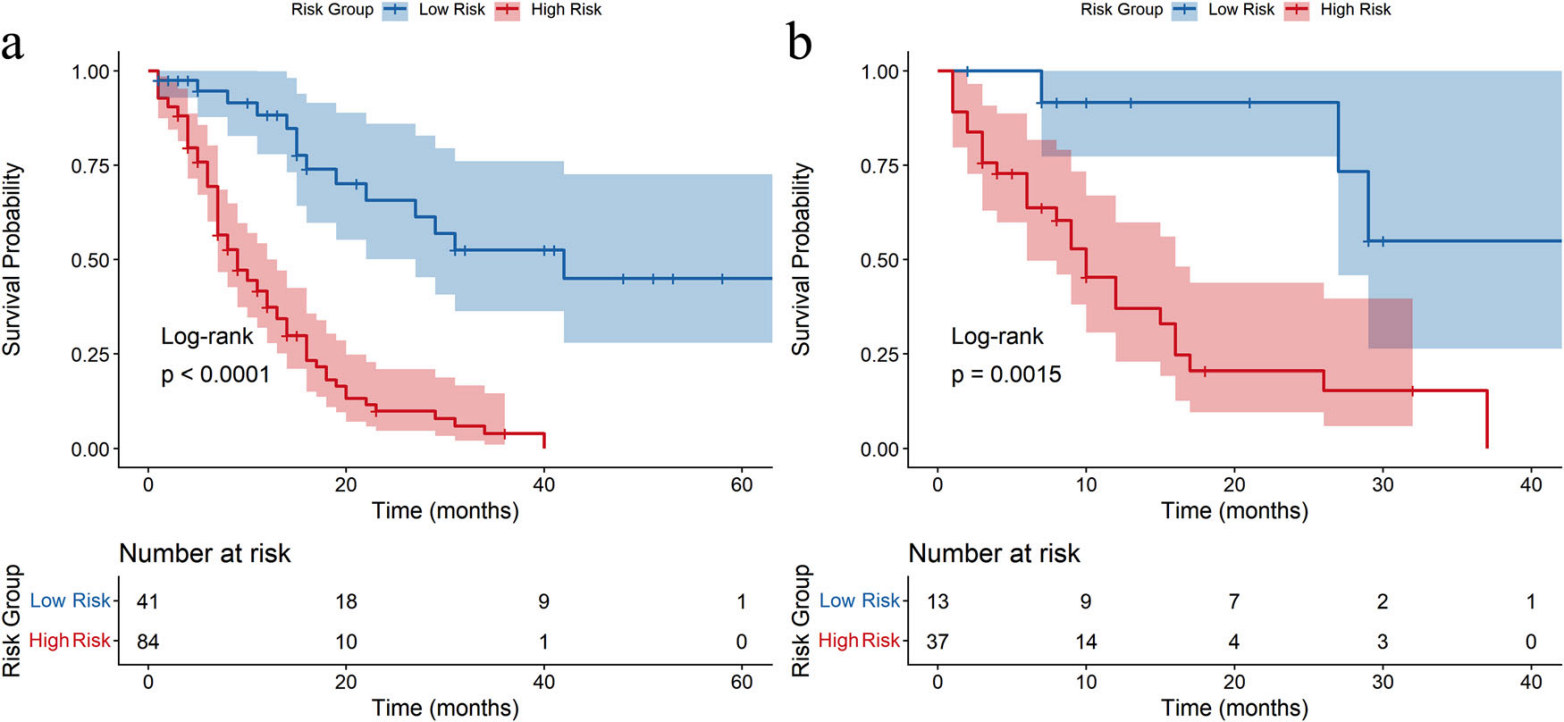
Kaplan–Meier curves showing the OS of IMCC patients stratified into predicted high- and low-risk groups in the training (**a**) and validation cohort (**b**). OS, overall survival

Using the Combined model, patients were stratified into high- and low-risk groups by an optimal cut-off automatically determined from the training set via the maximum Youden index and then fixed for the validation cohort. OS data were available for 175 out of 333 patients. In the overall OS-analysis cohort, the median overall survival was 18 months (95% CI: 12–29 months), with 112 deaths observed during a median follow-up of 32 months. The reasons for the unavailability of data for the remaining 158 patients were loss to follow-up (*n* = 72), recent diagnosis with insufficient follow-up time (*n* = 56), and incomplete medical records (*n* = 30). To assess potential bias, baseline characteristics were compared between the included and excluded groups, with no significant differences observed (all *p* > 0.05). As shown in Fig. 6, in the training cohort, high-risk patients had shorter OS than low-risk patients (median 9.000 [95% CI 8.000–12.000] vs 42.000 [95% CI 29.000–not reached (NR)] months; log-rank *p* < 0.001; HR 5.450 [95% CI 2.950–10.070], *p* < 0.001). In the validation cohort, results were consistent (10.000 [95% CI 9.000–16.000] vs NR [95% CI 29.000–NR] months; log-rank *p* = 0.002; HR 5.670 [95% CI 1.700–18.950], *p* = 0.005). In the overall OS-analysis cohort, high risk showed inferior survival (9.000 [95% CI 8.000–12.000] vs 42.000 [95% CI 29.000–NR] months; log-rank *p* < 0.001; HR 5.550 [95% CI 3.210–9.580], *p* < 0.001).

## Discussion

This study developed a DL model integrating complementary 2.5D and 3D multidimensional MRI biomarkers to improve noninvasive preoperative histologic grading of IMCC, with good external validation performance and biologically interpretable model outputs. In addition, the model showed preliminary prognostic relevance in the available OS-analysis cohort, suggesting its potential to provide supportive risk information for individualized preoperative assessment.

Building on this foundation, our research developed an interpretable ensemble DL framework that provides additional clinical insights to support treatment strategy optimization. The capacity to detect high-risk pathological grades [29] empowers physicians to administer targeted interventions earlier, such as neoadjuvant chemotherapy [30] or radical surgical procedures. Additionally, our DL framework may complement invasive biopsies by providing noninvasive grading information [31], particularly when a biopsy is contraindicated or yields inconclusive results, but it cannot replace histopathologic assessment [32].

In addition to grading prediction, our model demonstrated preliminary prognostic relevance in the available OS-analysis cohort. The observed survival stratification aligns with underlying pathological differences between risk groups. High-risk patients, as predicted by the model, likely harbor tumors with higher histological grades, which are typically associated with lower cellular differentiation, increased mitotic activity, and greater intratumoral heterogeneity. These pathological characteristics have been linked to more aggressive behavior and poorer prognosis across tumor types [3, 33]. These findings suggest that MRI-derived biomarker signatures for grading may also capture prognostically relevant tumor biology [34]. However, because OS data were available for only 175 of 333 patients, the survival-related results should be interpreted as exploratory and require prospective validation.

Previous radiomics and single-architecture DL studies in IMCC and related biliary tumors have mainly focused on handcrafted features [35], single-scale imaging representations, or diagnostic grading without systematic survival validation [36]. Important knowledge gaps remain regarding multiparametric MRI fusion, transparent model interpretability linking imaging to histopathology and prognosis, and robust external validation across centers. Xing et al developed DWI-based radiomic models with multiple machine learning classifiers but reported limited generalizability due to small cohorts and reliance on handcrafted features [36]. More recently, Fiz et al [37] showed that combined tumoral and peritumoral PET/CT radiomics improved the prediction of pathological grade and prognosis. However, radiomics models depend on pre-defined handcrafted features [35, 37] and may underrepresent complex three-dimensional growth patterns and cross-sequence interactions. In parallel, most DL-based studies have predominantly employed single-scale architectures—either 2D/2.5D designs [38–40] that sacrifice tumor volumetric context or 3D approaches [41] that underutilize sequence-specific signatures. Our study extends previous radiomics and single-architecture DL studies in IMCC by proposing an interpretable ensemble DL framework that synergizes 2.5D and 3D architectures with radiomics and ClinLabImag features. Our dual-scale DL approach learns hierarchical representations directly from MRI, thereby better capturing tumor-wide biological heterogeneity. This architectural synergy enables effective cross-scale tumor decoding and multimodal integration, offering complementary strengths for IMCC grading and exploratory prognostic assessment [38–41] compared with previous single-architecture or radiomics-based models [40, 42]. In parallel, recent transformer-based and DL radiomics models for cholangiocarcinoma and liver cancer have further emphasized the promise of attention mechanisms and hybrid architectures for tumor characterization [19, 20]. Many of these approaches, however, primarily rely on CT or ultrasound and focus on architectural innovation [43], whereas our framework provides complementary strengths by leveraging multiparametric MRI, dual-scale DL, radiomics, and ClinLabImag features together with SHAP and Grad-CAM interpretability and exploratory prognostic relevance. As such, our work contributes to the growing application of hybrid DL approaches in hepatobiliary oncology by focusing on IMCC-specific grading and prognosis using multiparametric MRI.

Tumor size was identified as the most robust imaging predictor of high-grade IMCC in this study, potentially reflecting enhanced proliferative activity, hypoxia, and neovascularization—hallmarks commonly associated with poor differentiation [29]. Infiltrative tumor margins also demonstrated a strong association with high-grade pathology, likely corresponding to underlying histological features such as stromal desmoplasia and loss of intercellular adhesion, particularly prominent in the large-duct subtype of IMCC [44]. Notably, vascular involvement was inversely correlated with tumor grade, which may suggest that highly aggressive tumors tend to disseminate via microscopic lymphovascular or perineural pathways rather than forming overt macrovascular encasement detectable by conventional MRI [45]. Additionally, the presence of a targetoid pattern on T2WI, often corresponding to necrotic, mucinous, or hemorrhagic tumor centers, may represent regions of low perfusion or high cellular density—a hallmark of tumor dedifferentiation [46]. These imaging biomarkers not only reflect underlying tumor biology but also offer accessible tools for noninvasive grading stratification.

These imaging biomarkers are further supported by Grad-CAM–based visualization. The Grad-CAM reflects biologically grounded tumor characteristics. DWI attention areas (high signal) align with histologically confirmed increases in tumor cellular density, a hallmark of high-grade malignancies where packed cells restrict water diffusion [47]. T2WI activation regions (hyperintensity) correspond to necrosis and mucin-rich stroma—features associated with aggressive phenotypes due to hypoxic microenvironments promoting angiogenesis [48]. This concordance between imaging biomarkers and pathobiology underscores our model’s capacity to decode biologically meaningful tumor patterns.

The SHAP analysis not only confirmed the predictive dominance of DL features but also revealed the underlying reasons for their superior contribution compared with radiomics and ClinLabImag variables. Unlike handcrafted radiomic descriptors or tabular clinical-laboratory indices, DL features capture multiscale representations [49] of intratumoral heterogeneity. These hierarchical image embeddings encode subtle spatial cues—such as microstructural irregularity, boundary infiltration, and texture anisotropy—that are often lost during manual feature engineering [50]. Such biologically meaningful patterns are closely associated with histologic differentiation, stromal desmoplasia, and tumor cellularity, thereby explaining the stronger SHAP attributions of DL-derived features. Several selected radiomics features, particularly texture-based and diffusion-related descriptors from T2WI and DWI sequences, align with known imaging biomarkers of intratumoral heterogeneity, stromal fibrosis, and diffusion restriction in high-grade IMCC. These features, including measures of gray-level nonuniformity, zone variance, and long-run emphasis, reflect the underlying biological characteristics such as tumor aggression, fibrosis, and cellularity that are commonly observed in high-grade tumors [51]. By quantitatively decomposing the model’s decision process, SHAP visualizations bridge the gap between algorithmic reasoning and radiologic interpretation, offering pathophysiologically grounded explanations.

Significant inter-cohort variability persisted in sex, CA-125, and imaging parameters, a common challenge in multicenter studies [52]. These discrepancies likely arise from center-specific differences in patient case-mix, laboratory assays, and MRI acquisition protocols, introducing domain shifts that both challenge and more realistically evaluate the model’s generalizability [53]. Nevertheless, we recognize that the model demonstrated reduced sensitivity for identifying high-grade tumors in the external validation cohort. This discrepancy may arise from multiple factors: (1) domain shift introduced by inter-center variability in imaging protocols and scanner parameters; (2) class imbalance, with fewer high-grade samples potentially biasing the model’s decision boundary; and (3) histological–radiological discordance in mucin-rich or heterogeneous tumors, which may confound radiologic prediction [7]. The higher training AUC of the DL model (0.978 vs 0.804 external) likely reflects in-sample optimism and cross-center domain shift rather than overfitting alone; thus, external AUC provides a more realistic estimate of generalizability. Although the Combined model achieved a numerically higher AUC (0.843 [95% CI: 0.749–0.938]) than the DL model (0.804 [95% CI: 0.712–0.896]) in external validation (DeLong *p* = 0.355), the incremental value of adding radiomics and ClinLabImag features remained modest. These findings highlight the complementary role of multimodal integration in offering additional clinical context and interpretability without compromising the core DL framework’s robust performance.

In clinical practice, the proposed framework has the potential to serve as a noninvasive decision-support tool for histologic grading and preliminary OS-related risk assessment in challenging IMCC cases, particularly when biopsy is contraindicated or yields inconclusive results. The present retrospective study provides evidence that an interpretable ensemble DL model based on multidimensional MRI can be externally validated and associated with exploratory OS information in a multicenter setting. However, given the retrospective design and manual tumor segmentation, the current evidence does not address real-world workflow integration, cost-effectiveness, or direct impact on clinical decision-making. Future studies are needed to explore these aspects. Future work will prospectively evaluate this framework in larger, more balanced multicenter cohorts and explore its integration into clinical workstations to provide real-time decision support for individualized preoperative management in IMCC.

## Limitations

Our study has several limitations. First, despite prespecified eligibility criteria and external validation, the retrospective multicenter design may still introduce selection bias and unmeasured confounding, and generalizability to rare histologic variants remains uncertain. Second, the model may be less reliable in lesions with extensive necrosis, hemorrhage, or mucin-rich components, causing ambiguous DWI/T2WI signals; therefore, predictions should be interpreted cautiously and integrated with biopsy findings and multidisciplinary judgment. Third, the relatively small external validation cohort may have reduced sensitivity for high-grade disease and limited the precision of performance estimates. Fourth, cross-center acquisition heterogeneity may still affect the stability and external applicability of radiomics and deep learning features. Formal subgroup and biopsy-based concordance analyses were not performed because of the retrospective design, small subgroup sizes, and limited standardized biopsy data. We further acknowledge that the marked performance gap between training (AUC 0.978) and external validation (AUC 0.804 for the DL model) primarily reflects in-sample optimism and cross-center domain shift rather than uncontrolled overfitting alone. This remains an important limitation of the current study. Finally, the survival analysis is exploratory and should be interpreted cautiously because follow-up was limited or incomplete in some patients.

## Conclusion

In conclusion, we developed an interpretable multimodal framework integrating multidimensional MRI, radiomics, and ClinLabImag features for noninvasive preoperative grading of IMCC. The Combined model demonstrated good external validation performance and showed preliminary prognostic relevance.

## Supplementary information

Supplementary information

## Data Availability

The data used and analyzed during the current study are available from the corresponding authors on reasonable request.

## Abbreviations

ASLSingleLabel: Asymmetric loss function single label
AUC: Area under the receiver operating characteristic curve
CA-125: Carbohydrate antigen 125
ClinLabImag: Clinical-laboratory-imaging
DL: Deep learning
DWI: Diffusion-weighted imaging
Grad-CAM: Gradient-weighted Class Activation Mapping
IMCC: Intrahepatic mass-forming cholangiocarcinoma
NRI: Net reclassification improvement
OS: Overall survival
SHAP: SHapley Additive exPlanations
T2WI: T2-weighted imaging
VOI: Volumes of interest
WHO: World Health Organization
