# Supplementary material for: Multidimensional deep learning for grading and prognostic assessment of intrahepatic mass-forming cholangiocarcinoma
Source: Insights Imaging. 2026 Jul 22;17:187. doi: 10.1186/s13244-026-02350-0 (PMC13391990; doi:10.1186/s13244-026-02350-0)
Supplement: Supplementary file 1 — Supplementary information [file 13244_2026_2350_MOESM1_ESM.pdf]

Multidimensional Deep Learning for Grading and Prognostic Assessment of Intrahepatic Mass-Forming Cholangiocarcinoma

Electronic supplementary material

Section 1 Definition of Qualitative Imaging Features

Imaging features were defined as: (1) Targetoid sign - hyperintense peripheral ring or central area; (2) Tumor thrombus - vascular invasion by neoplastic tissue; (3) Vascular traversal - vessels penetrating tumor; (4) Intrahepatic duct dilatation - abrupt biliary dilation with T2WI hyperintensity[18]; (5) Capsular retraction - liver surface indentation near tumor; (6) Enlarged lymph nodes - ≥1 cm short-axis diameter[25]; (7) Tumor size - the maximum diameter of the lesion measured on axial T2WI. Following independent assessments, interobserver agreement was quantified using intraclass correlation coefficients (rang: 0.83-0.95), demonstrating substantial-to-excellent consistency.

Section 2 Tumor segmentation and Reproducibility Analysis

Two radiologists (radiologist A: 10 years; radiologist B: 5 years of abdominal MRI experience) manually segmented tumor regions on T2WI and DWI using ITK-SNAP software (version 4.0.0). Segmentation employed free-hand contouring with subsequent ITK-SNAP algorithmic smoothing and interpolation to ensure volumetric accuracy. Consensus-derived volume of interest (VOI) was validated by a third radiologist (L.X., >15 years' experience). To assess reproducibility, 20 randomly selected cases from the training cohort were independently segmented by both observers, and radiologist A repeated the segmentation after a four-week interval. The mean inter-observer Dice similarity coefficient (DSC) was 0.86 ± 0.05, while the intra-observer DSC was 0.89 ± 0.04. For volumetric assessment, the inter-observer intraclass correlation coefficient was 0.92 (95% CI: 0.88 – 0.95) and the intra-observer intraclass correlation coefficient was 0.94 (95% CI: 0.91 – 0.97), indicating excellent agreement according to the established thresholds.

Section 3 Image Preprocessing

All MRI volumes were resampled to isotropic $1 \times 1 \times 8$ mm^3 voxel spacing using third-order B-spline interpolation. For the 2.5D branch, seven consecutive axial slices centred on the tumour-containing slice were selected (determined as the cohort median number of tumour-containing slices), cropped to 128×128 pixels with 10-pixel perilesional expansion, truncated at the 1st and 99th intensity percentiles, and min-max normalized. For the 3D branch, VOIs were isotropically expanded by 10 voxels, cropped to $128 \times 128 \times 32$ voxels, and underwent identical intensity normalization. Zero-padding was applied when required.

Section 4 Detailed Construction of ClinLabImag Model

All clinical, laboratory, and qualitative imaging variables listed in Table 1 were initially screened by univariable logistic regression (high-grade vs. low-grade as dependent variable). Variables with $P<0.05$ were retained as candidates. These four candidate variables were then entered into a backward stepwise multivariable logistic regression (entry $P<0.05$, removal $P>0.10$). The final ClinLabImag model retained four independent predictors: tumor size, tumor margin, vascular involvement, and T2WI signal pattern.

Section 5 Detailed Radiomics Feature Extraction and Model Construction (including filter list)

Images underwent N4 bias-field correction, resampling to $1 \times 1 \times 8$ mm^3 [26], and intensity normalization. VOIs were registered between sequences using hybrid affine and deformable transformations[27]. A total of 4,528 radiomic features were extracted from T2WI and DWI sequences combined (2,264 features per sequence) using PyRadiomics v3.0[28,29]. Per sequence, the feature set consisted of 14 three-dimensional morphological (shape) features, 18 first-order intensity statistics, and 72 textural features (24 gray-level co-occurrence matrix [GLCM], 16 gray-level run length matrix [GLRLM], 16 gray-level size zone matrix [GLSZM], 14 gray-level dependence matrix [GLDM], and 5 neighboring gray-tone difference matrix [NGTDM]) derived from the original images. In addition, 2,160 higher-order features were generated by applying 24 image filters, comprising 432 filtered first-order

features (18 × 24) and 1,728 filtered textural features (72 × 24). Thus, the complete breakdown per sequence was 14 morphological + 450 first-order (18 original + 432 filtered) + 1,800 textural (72 original + 1,728 filtered) = 2,264 features. Feature selection was performed using chi-square test followed by least absolute shrinkage and selection operator (LASSO) regression. We adopted LASSO for feature selection because its L1 regularization is well suited to high-dimensional, collinear radiomics spaces and has been widely validated in oncologic radiomics studies.

MR Image Filters List

1. Additive Gaussiannoise Filter

Alter an image with additive Gaussian white noise.Additive Gaussian white noise can be modeled as:

$$I = I_0 + N$$

Where I is the observed image, I_0 is the noise-free image and N is a normally distributed random variable of mean σ^2 and variance :

$$N \sim N(\mu, \sigma^2)$$

The noise is independent of the pixel intensities.

2. Bilateral Filter

This filter uses bilateral filtering to blur an image using both domain and range "neighborhoods". Pixels that are close to a pixel in the image domain and similar to a pixel in the image range are used to calculate the filtered value. Two gaussian kernels (one in the image domain and one in the image range) are used to smooth the image. The result is an image that is smoothed in homogeneous regions yet has edges preserved. The result is similar to anisotropic diffusion but the implementation in non-iterative. Another benefit to bilateral filtering is that any distance metric can be used for kernel smoothing the image range. Hence, color images can be smoothed as vector images, using the CIE distances between intensity values as the similarity metric (the Gaussian kernel for the image domain is evaluated using CIE distances). A separate version of this filter will be designed for color and vector images.Bilateral filtering is capable of reducing the noise in

an image by an order of magnitude while maintaining edges.

3. Binomial Blur Image Filter

Performs a separable blur on each dimension of an image.The binomial blur consists of a nearest neighbor average along each image dimension. The net result after n-iterations approaches convultion with a gaussian.

4. Box Mean Filter

Implements a fast rectangular mean filter using the accumulator approach.

5. Box Sigma Image Filter

Implements a fast rectangular sigma filter using the accumulator approach.

6. Curvature Flow Filter

Denoise an image using curvature driven flow.CurvatureFlowImageFilter implements a curvature driven image denoising algorithm. Iso-brightness contours in the grayscale input image are viewed as a level set. The level set is then evolved using a curvature-based speed function:

$$I_k =_k \backslash \nabla I \backslash$$

where κ is the curvature.

The advantage of this approach is that sharp boundaries are preserved with smoothing occurring only within a region. However, it should be noted that continuous application of this scheme will result in the eventual removal of all information as each contour shrinks to zero and disappear.

Note that unlike level set segmentation algorithms, the image to be denoised is already the level set and can be set directly as the input using the SetInput() method.

This filter has two parameters: the number of update iterations to be performed and the timestep between each update.

The timestep should be "small enough" to ensure numerical stability. Stability is guarantee when the timestep meets the CFL (Courant-Friedrichs-Levy) condition. Broadly speaking, this condition ensures that each contour does not move more than one grid position at each timestep. In the literature, the timestep is typically user specified and have to manually tuned to the application.

This filter make use of the multi-threaded finite difference solver hierarchy. Updates are computed using a Curvature Flow Function object. A zero flux Neumann boundary condition when computing derivatives near the data boundary. This filter may be streamed. To support streaming this filter produces a padded output which takes into account edge effects. The size of the padding is m_NumberOfIterations on each edge. Users of this filter should only make use of the center valid central region.

7. Discrete Gaussian Filter

Blurs an image by separable convolution with discrete gaussian kernels. This filter performs Gaussian blurring by separable convolution of an image and a discrete Gaussian operator (kernel).The Gaussian operator used here was described by Tony Lindeberg (Discrete Scale-Space Theory and the Scale-Space Primal Sketch. Dissertation. Royal Institute of Technology, Stockholm, Sweden. May 1991.) The Gaussian kernel used here was designed so that smoothing and derivative operations commute after discretization.

8. Laplacian Sharpening Filter

This filter sharpens an image using a Laplacian. LaplacianSharpening highlights regions of rapid intensity change and therefore highlights or enhances the edges. The result is an image that appears more in focus.

9. Mean Filter

Applies an averaging filter to an image.Computes an image where a given pixel is the mean value of the the pixels in a neighborhood about the corresponding input pixel.A mean filter is one of the family of linear filters.

10. Median Filter

Applies a median filter to an image.Computes an image where a given pixel is the median value of the the pixels in a neighborhood about the corresponding input pixel.A median filter is one of the family of nonlinear filters. It is used to smooth an image without being biased by outliers or shot noise.

11. Normalize Filter

Normalize an image by setting its mean to zero and variance to one.

NormalizeImageFilter shifts and scales an image so that the pixels in the image have a zero mean and unit variance. This filter uses StatisticsImageFilter to compute the mean and variance of the input and then applies ShiftScaleImageFilter to shift and scale the pixels.NB: since this filter normalizes the data to lie within -1 to 1, integral types will produce an image that DOES NOT HAVE a unit variance.

12. Recursive Gaussian Filter

Base class for computing IIR convolution with an approximation of a Gaussian kernel.

$$\frac{1}{\sigma\sqrt{2\pi}}exp(-\frac{x^2}{2\sigma^2})$$

RecursiveGaussianImageFilter is the base class for recursive filters that approximate convolution with the Gaussian kernel. This class implements the recursive filtering method proposed by R.Deriche in IEEE-PAMI Vol.12, No.1, January 1990, pp 78-87, "Fast Algorithms for Low-Level Vision"

13. Shot Noise Filter

Alter an image with shot noise.The shot noise follows a Poisson distribution:

$$I = N(I_0)$$

where $N(I_0)$ is a Poisson-distributed random variable of mean I_0. The noise is thus dependent on the pixel intensities in the image.

The intensities in the image can be scaled by a user provided value to map pixel values to the actual number of particles. The scaling can be seen as the inverse of the gain used during the acquisition. The noisy signal is then scaled back to its input intensity range:

$$I = \frac{N(I_0 \times \delta)}{\delta}$$

where δ is the scale factor.

The Poisson-distributed variable λ is computed by using the

$$k \leftarrow 0$$
$$p \leftarrow 1$$
algorithm: $$\text{repeat}\begin{cases} k = k + 1 \\ p = p * U() \end{cases}$$
$$until p > e^{\lambda}, reture(k)$$

where U() provides a uniformly distributed random variable in the interval [0,1] . This algorithm is very inefficient for large values of λ , though. Fortunately, the Poisson distribution can be accurately approximated by a Gaussian distribution of mean and variance λ when λ is large enough. In this implementation, this value is considered to be 50. This leads to the faster algorithm:

$$\lambda + \sqrt{\lambda} \times N()$$

where $N()$ is a normally distributed random variable of mean 0 and variance 1.

14. Smoothing Recursive Gaussian Filter

Computes the smoothing of an image by convolution with the Gaussian kernels implemented as IIR filters.This filter is implemented using the recursive gaussian filters. For multi-component images, the filter works on each component independently.For this filter to be able to run in-place the input and output image types need to be the same and/or the same type as the RealImageType.

15. Speckle Noise Filter

Alter an image with speckle (multiplicative) noise.The speckle noise follows a gamma distribution of mean 1 and standard deviation provided by the user. The noise is proportional to the pixel intensity.

It can be modeled as:

$$I = I_0 * G$$

where $G$is a is a gamma distributed random variable of mean 1 and variance proportional to the noise level:

$$G \sim \Gamma(\frac{1}{\sigma^2}, \sigma^2)$$

16.LoGFilter

Computes the Laplacian of Gaussian (LoG) of an image.Computesthe Laplacian of

Gaussian(LoG) of an image by convolution with the second derivative of a Gaussian. This filter is implemented using the recursive gaussian filters.

17.WaveletFilter

The WaveletFilter decomposition employs **8 distinct sub-filters** generated through parameterized scaling functions. A wavelet is a wave-like oscillation with an amplitude that begins at zero, increases, and then decreases back to zero. It can typically be visualized as a "brief oscillation" like one recorded by a seismograph or heart monitor. Generally, wavelets are intentionally crafted to have specific properties that make them useful for signal processing. Using a "reverse, shift, multiply and integrate" technique called convolution, wavelets can be combined with known portions of a damaged signal to extract information from the unknown portions.

For example, a wavelet could be created to have a frequency of Middle C and a short duration of roughly a 32nd note. If this wavelet were to be convolved with a signal created from the recording of a song, then the resulting signal would be useful for determining when the Middle C note was being played in the song. Mathematically, the wavelet will correlate with the signal if the unknown signal contains information of similar frequency. This concept of correlation is at the core of many practical applications of wavelet theory.

As a mathematical tool, wavelets can be used to extract information from many different kinds of data, including – but not limited to – audio signals and images. Sets of wavelets are generally needed to analyze data fully. A set of "complementary" wavelets will decompose data without gaps or overlap so that the decomposition process is mathematically reversible. Thus, sets of complementary wavelets are useful in wavelet based compression/decompression algorithms where it is desirable to recover the original information with minimal loss.

In formal terms, this representation is a wavelet series representation of a square-integrable function with respect to either a complete, orthonormal set of basis functions, or an overcomplete set or frame of a vector space, for the Hilbert space of square integrable functions. This is accomplished through coherent states.

Section 6 Expert Evaluation of Grad-CAM－VOI Overlap

To qualitatively and semi-quantitatively assess the spatial correspondence between Grad-CAM attention maps and the tumor regions used for model training, we performed an expert overlap evaluation using the manually delineated volumes of interest (VOIs) on T2WI and DWI. The VOIs were identical to those employed for radiomics extraction and deep learning model development.

Random 20 cases covering both low-grade and high-grade IMCC from the training and external validation cohorts was selected for this analysis. For each selected case, Grad-CAM saliency maps were generated for the 2.5D and 3D branches on both T2WI and DWI, as described in the main text (see Figure 2 for representative examples). High-activation regions were overlaid on the corresponding MR images and VOIs.

Two abdominal radiologists (each with 10 years of experience in liver MRI), blinded to the model outputs, clinical information, and histopathology, independently rated the degree of spatial overlap between the Grad-CAM high-activation regions and the tumor VOIs. Overlap was scored using a 5-point Likert scale: 1 = minimal or no overlap, 2 = slight overlap, 3 = partial overlap, 4 = substantial overlap, and 5 = near-complete overlap between Grad-CAM activation and the VOI. Scores were recorded separately for T2WI and DWI, and for the 2.5D and 3D Grad-CAM maps where applicable.

For Reader 1, the mean overlap score was 4.20, with a median of 4.0, an interquartile range (IQR) of 4.0–5.0, and a range of 3–5; 85% (17/20) of the ratings were ≥4. For Reader 2, the mean score was 4.05, with a median of 4.0, an IQR of 3.75–5.0, and a range of 2–5; 75% (15/20) of the ratings were ≥4. Considering all 40 ratings from both readers combined, the overall mean score was 4.13, with a median of 4.0, an IQR of 4.0–5.0, and a range of 2–5, and 80% of ratings were ≥4, indicating that Grad-CAM activations generally focused on regions overlapping well with the tumor VOIs. Inter-reader agreement was high: the two radiologists provided identical scores in 85% (17/20) of cases, and the quadratic weighted Cohen’s kappa was 0.88, consistent with excellent agreement.

Table S1 MRI Acquisition Parameters for the Training and Test Datasets

Dataset	Scanner	Sequence	TR/ TE (ms)	FOV (mm)	Matrix	Sections Thickness (mm)	Section Gap (mm)	No. of Sections	Flip Angle
Training dataset	GE 3.0T (Discovery MR 750)	T2WI	(5000-8000)/(86-100)	(400, 300)	(320, 320)	5	2.5	2	120
		DWI	(3000-7500)/(46-66)	(380, 300)	(128, 130)	5	1	1	90
	UI 1.5T (uMR 588)	T2WI	(4500-6700)/(80-90)	(350, 350)	(320, 320)	5	2	2	150
		DWI	(3085-7060)/(45-70)	(380, 300)	(128, 128)	6	1	1	90
	Siemens 1.5T (Amira)	T2WI	(4099-5187)/(89-102)	(350, 350)	(320, 288)	5	2	2	100
		DWI	(3120-7000)/(45-70)	(380, 300)	(380, 250)	6	1	1	90
External test dataset	GE 1.5T (Optima MR360)	T2WI	(4099-5187)/(89-102)	(350, 350)	(288, 288)	5	2	1	150, 120
		DWI	(3120-7000)/(45-70)	(380, 300)	(140, 140)	6	1	1	90
	GE 3.0T (Signa HDx)	T2WI	(3000-6000)/(67.6-85)	(350, 350)	(320, 224)	5-6	2	0.5	100,145
		DWI	(7100-9230)/(66-	(400, 320)	(128, 128)	6	1	1	90

	79.6)

FOV, field of view, TR, repetition time, TE, echo time, No., number, ms, millisecond, mm, millimeter

Table S2 The 24 final selected radiomic features.

No.	Feature Name	Category	Filter Origin	Sequence Source
1	T2_FirstOrder_Mean	First-order	Original	T2WI
2	T2_GLCM_Correlation	Textural	Original	T2WI
3	T2_Shape_SurfaceArea	Morphological	Original	T2WI
4	T2_GLRLM_ShortRunEmphasis	Textural	LoG (σ=2.0)	T2WI
5	T2_FirstOrder_Kurtosis	First-order	Wavelet (HLH)	T2WI
6	DWI_GLCM_Contrast	Textural	Wavelet (LLH)	DWI
7	DWI_FirstOrder_Skewness	First-order	Original	DWI
8	DWI_Shape_Compactness	Morphological	Original	DWI
9	T2_GLSZM_GrayLevelVariance	Textural	Square root	T2WI
10	DWI_GLCM_Energy	Textural	LoG (σ=3.0)	DWI
11	T2_GLRLM_RunEntropy	Textural	Wavelet (HHL)	T2WI
12	DWI_GLDZM_SizeZoneVariance	Textural	Exponential	DWI
13	T2_FirstOrder_Entropy	First-order	Wavelet (LLL)	T2WI
14	T2_GLCM_Dissimilarity	Textural	Logarithm	T2WI
15	DWI_FirstOrder_Uniformity	First-order	Original	DWI
16	DWI_GLRLM_LongRunEmphasis	Textural	Smoothing	DWI
17	T2_GLCM_ClusterShade	Textural	Wavelet (LHL)	T2WI
18	T2_FirstOrder_Maximum	First-order	Original	T2WI
19	DWI_GLSZM_ZoneEntropy	Textural	Wavelet (HLH)	DWI
20	T2_FirstOrder_Variance	First-order	Gradient	T2WI
21	DWI_Shape_Sphericity	Morphological	Original	DWI

22	T2_GLCM_Autocorrelation	Textural	LoG (σ=4.0)	T2WI
23	DWI_FirstOrder_Median	First-order	Wavelet (HHH)	DWI
24	T2_GLRLM_RunLengthNonUniformity	Textural	Exponential	T2WI

Table S3 Checklist for Artificial Intelligence in Medical Imaging (CLAIM)

Section / Topic	No.	Item	Page / Line	No	NA
TITLE / ABSTRACT					
	**1**	Identification as a study of AI methodology, specifying the category of technology used (e.g., deep learning)	**1/1**		
ABSTRACT					
	**2**	Summary of study design, methods, results, and conclusions	**1/2**		
INTRODUCTION					
	**3**	Scientific and/or clinical background, including the intended use and role of the AI approach	**3/1**		
	**4**	Study aims, objectives, and hypotheses	**4**		
METHODS					
Study Design	**5**	Prospective or retrospective study	**6/10**		
	**6**	Study goal	**6/13**		

Data	**7**	Data sources	**6/15**		
	**8**	Inclusion and exclusion criteria	**6/10**		
	**9**	Data pre-processing	**7/6**		
	**10**	Selection of data subsets	**7/10**		
	**11**	De-identification methods	**7/15**		
	**12**	How missing data were handled	**7/16**		
	**13**	Image acquisition protocol	**7/6**		
Reference Standard	**14**	Definition of method(s) used to obtain reference standard	**7/16**		
	**15**	Rationale for choosing the reference standard	**7/16**		
	**16**	Source of reference standard annotations	**7/16**		
	**17**	Annotation of test set	**7/16**		
	**18**	Measures of inter- and intra-rater variability of features described by the annotators	**7/16**		
Data Partitions	**19**	How data were assigned to partitions	**6/10**		
	**20**	Level at which partitions are disjoint	**6/10**		
Testing Data	**21**	Intended sample size	**6/10**		

Section / Topic	No.	Item	Page / Line	No	NA
Model	**22**	Detailed description of model	**8/8**		
	**23**	Software libraries, frameworks, and packages	**8/8**		
	**24**	Initialization of model parameters	**8/8**		
Training	**25**	Details of training approach	**8/8**		
	**26**	Method of selecting the final model	**8/8**		
	**27**	Ensembling techniques	**9/8**		
Evaluation	**28**	Metrics of model performance	**10/6**		
	**29**	Statistical measures of significance and uncertainty	**10/6**		
	**30**	Robustness or sensitivity analysis	**10/6**		
	**31**	Methods for explainability or interpretability	**10/6**		
	**32**	Evaluation on internal data	**10/6**		
	**33**	Testing on external data	**10/6**		
	**34**	Clinical trial registration	**10/6**		
RESULTS					
Data	**35**	Numbers of patients or examinations included and excluded	**10/18**		

	**36**	Demographic and clinical characteristics of cases in each partition	**10/18**		
Model performance	**37**	Performance metrics and measures of statistical uncertainty	**10/18**		
	**38**	Estimates of diagnostic performance and their precision	**10/18**		
	**39**	Failure analysis of incorrect results	**10/18**		
DISCUSSION					
	**40**	Study limitations	**20/19**		
	**41**	Implications for practice, including intended use and/or clinical role	**20/19**		
OTHER INFORMATION					
	**42**	Provide a reference to the full study protocol or to additional technical details	**Declaration file**		
	**43**	Statement about the availability of software, trained model, and/or data	**Declaration file**		
	**44**	Sources of funding and other support; role of funders	**Declaration file**		

Figure S1 Flowchart of the patient inclusion and exclusion process in multicenter IMCC study.

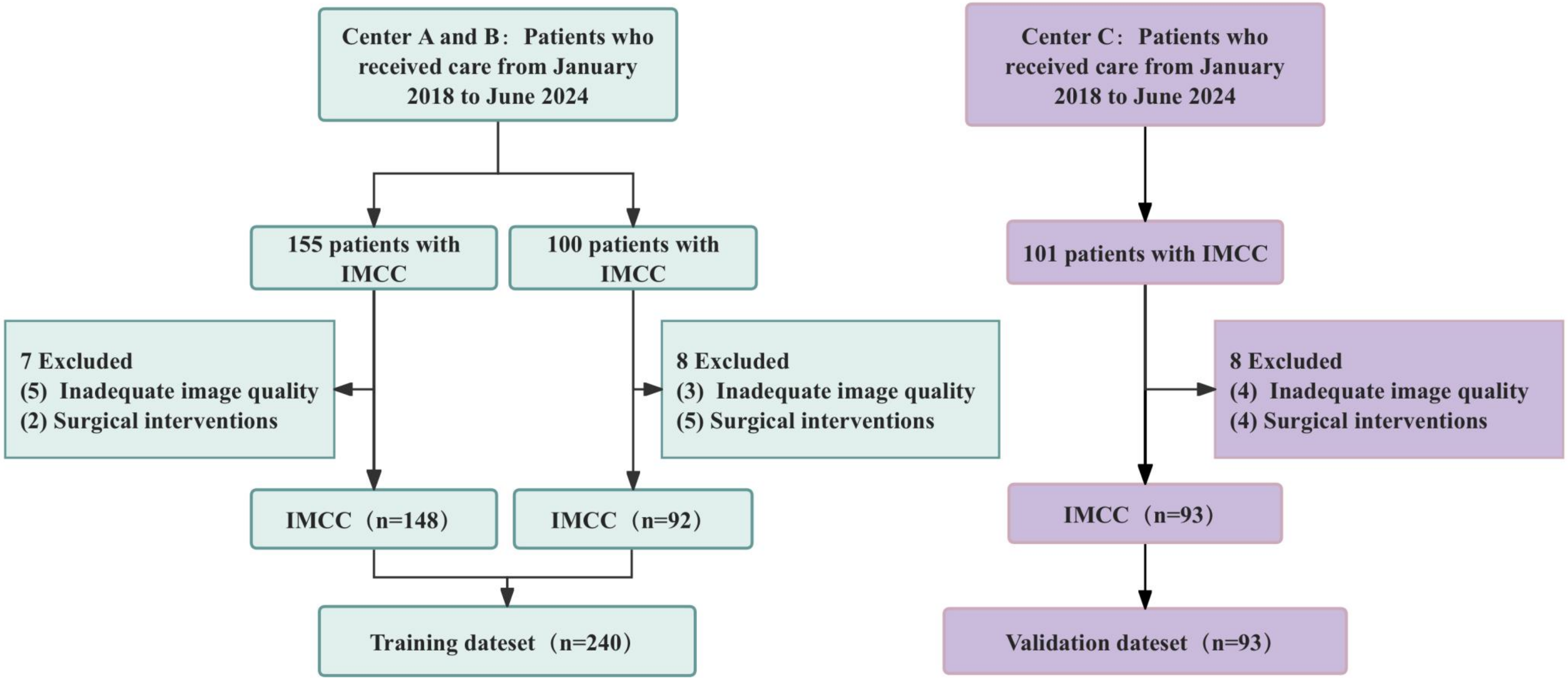

IMCC, intrahepatic mass-forming cholangiocarcinoma

Figure S2 Heatmap of top 10 deep learning – derived and radiomics features for prediction of IMCC histologic grade.

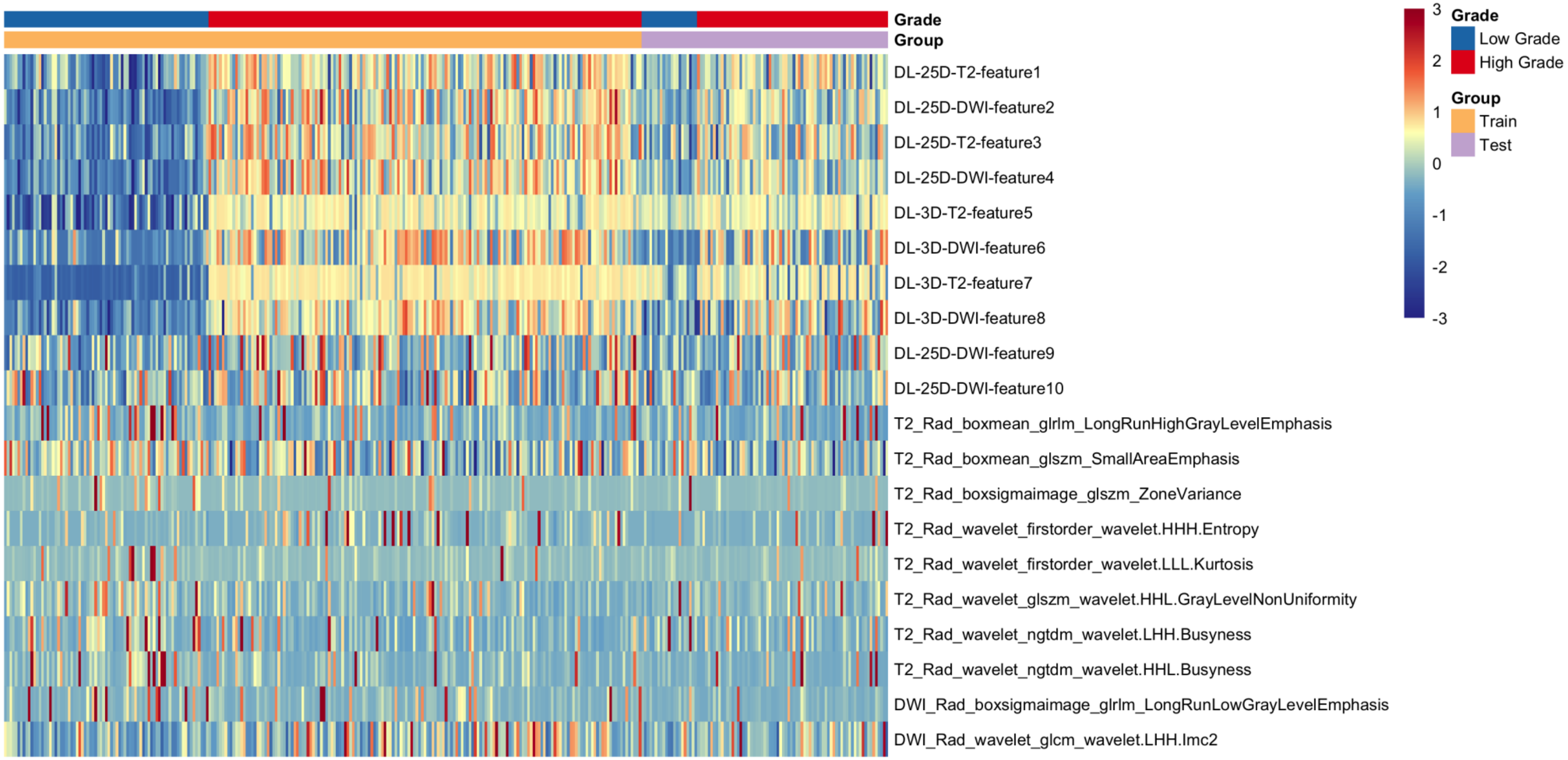

Rows represent the 10 most important deep learning – derived (DL-25D-T2, DL-25D-DWI, DL-3D-T2, DL-3D-DWI) and

handcrafted radiomics features extracted from T2-weighted and diffusion-weighted MR images that were used for prediction of IMCC histologic grade. Feature values were displayed with a diverging color scale (blue, lower than cohort mean; white, near mean; red, higher than cohort mean). The color bars at the top indicate histologic grade (Low Grade, High Grade) and data cohort (Train, Test) for each patient.

Figure S3 Local SHAP Explanations for IMCC Pathologic Grade Prediction

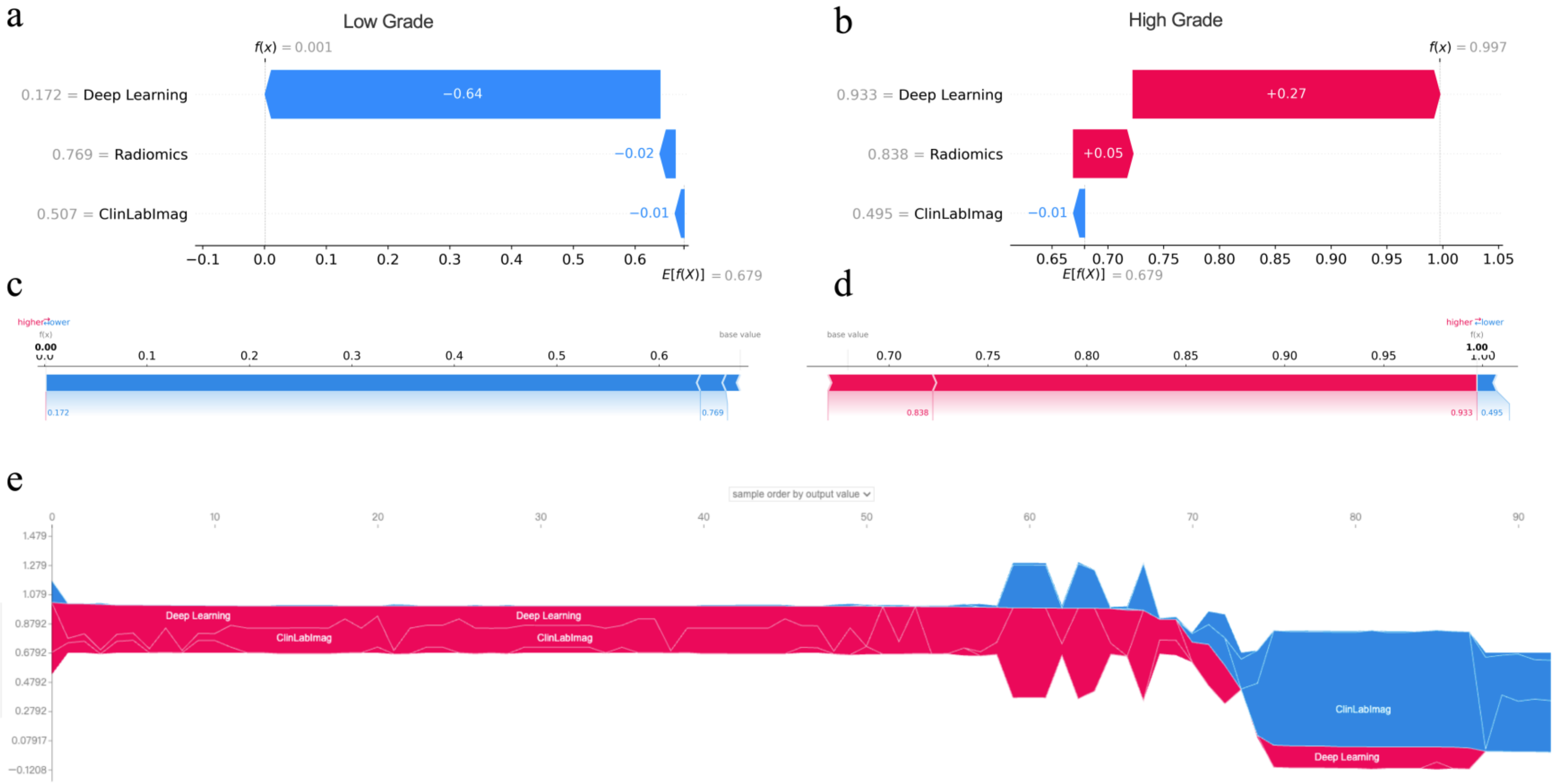

Panels (a) and (c) illustrate low-grade predictions, whereas panels (b) and (d) illustrate high-grade predictions. SHAP values quantify each feature’ s local contribution to the predicted probability of high grade (positive) or low grade (negative). Panel

(e) illustrates the feature contributions for each patient in the validation cohort. Each patient is plotted along the x-axis, while the magnitude and direction of feature contributions are represented by the red (high-grade) and blue (low-grade) segments; a larger red portion for an individual indicates a higher predicted grade.

ClinLabImag, Clinical-Laboratory-Imaging.
